# Design and evaluation of an imager for assessing wound inflammatory responses and bioburden in a pig model

**DOI:** 10.1117/1.JBO.25.3.032002

**Published:** 2019-09-12

**Authors:** Ashley Dacy, Nowmi Haider, Kathryn Davis, Wenjing Hu, Liping Tang

**Affiliations:** a University of Texas at Arlington, Department of Bioengineering, Arlington, Texas, United States; b University of Texas Southwestern Medical Center, Department of Plastic Surgery, Dallas, Texas, United States; c Progenitec Inc., Arlington, Texas, United States

**Keywords:** reactive oxygen species, luminescence, wound healing, vascularization, infection, nanoprobe

## Abstract

Our work details the development and characterization of a portable luminescence imaging device for detecting inflammatory responses and infection in skin wounds. The device includes a CCD camera and close-up lens integrated into a customizable 3D printed imaging chamber to create a portable light-tight imager for luminescence imaging. The chamber has an adjustable light portal that permits ample ambient light for white light imaging. This imager was used to quantify in real time the extent of two-dimensional reactive oxygen species (ROS) activity distribution using a porcine wound infection model. The imager was used to successfully visualize ROS-associated luminescent activities *in vitro* and *in vivo*. Using a pig full-thickness cutaneous wound model, we further demonstrate that this portable imager can detect the change of ROS activities and their relationship with vasculature in the wound environment. Finally, by analyzing ROS intensity and distribution, an imaging method was developed to distinguish infected from uninfected wounds. We discovered a distinct ROS pattern between bacteria-infected and control wounds corresponding to the microvasculature. The results presented demonstrate that this portable luminescence imager is capable of imaging ROS activities in cutaneous wounds in a large animal model, indicating suitability for future clinical applications.

## 1 Introduction

Wound care represents one of the fastest-growing segments of the modern healthcare market.^1^ In the United States, chronic wounds are estimated to cost $20 billion and affect over 6.5 million patients per year.^2^ Chronic, nonhealing wounds present complex treatment and diagnostic difficulties. It is well-established that inflammatory products, including reactive oxygen species (ROS), have critical influence over the healing process.^3^^–^^5^ However, traditional wound healing diagnostic techniques rely on wound size and volume measurements, which have been found to have poor accuracy.^6^^,^^7^ On the other hand, wound bacterial infection and infiltration (also referred to as bioburden) have been well established to delay wound healing.^8^ Wound infection diagnosis (typically via a swab culture) is time-consuming and requires access to a specialized facility.^9^^,^^10^ Therefore, there is a need for the development of techniques to provide real-time assessment of wound healing and infection/bioburden status, which is the overall goal of this investigation.

ROS plays a pivotal role in the orchestration of wound healing responses. Specifically, ROS and their associated radicals have been implicated as important intracellular second messengers at low concentrations, mediating responses such as ATP production.^11^ ROS gradients also interact with platelets to facilitate thrombus formation after wounding.^12^ In addition, ROS is a key factor in triggering cell division and migration in fibroblasts, keratinocytes, and endothelial cells.^13^^,^^14^ Critically, ROS is also produced by migrating inflammatory cells. It has been shown that vascularity has a strong connection with inflammation, inflammatory cell infiltration, and wound ROS levels. Specifically, inflammatory cells migrate along the endothelial surface and through postcapillary venules (25- to 50-μm diameter^15^) into the wound.^16^ These cells release a large amount of ROS into phagosomes to kill engulfed bacteria as part of the inflammatory healing phase.^5^^,^^17^^,^^18^ Macrophages recruited ∼2 days postwounding have also been shown to produce ROS.^19^ In chronic wounds, excessive ROS released in wounds can delay healing and cause tissue damage.^3^^,^^12^^,^^20^^,^^21^ Reducing ROS levels in chronic mouse wounds using antioxidants has been shown to move wounds out of the chronic cycle and into a healthy healing process.^22^ ROS has been found to modulate angiogenesis by inducing expression of the proangiogenic growth factor VEGF in keratinocytes and macrophages.^5^^,^^23^ Studies have also shown that ROS is a key regulator of vasorelaxation and inflammatory cell adhesion in blood vessel walls.^24^ ROS gradients have been suggested to promote endothelial cell growth and migration at concentrations as low as 100  μM.^12^^,^^25^ ROS also plays a critical role in wound infection and bacteria detection. Equally important, ROS levels may be increased by an order of magnitude in an infected wound.^26^ Treatment of infected wounds with ROS-reducing antioxidants has been shown to significantly decrease wound bacterial bioburden.^22^ Due to its many roles in healing, vascularization, and correlation to infection and chronicity in wounds, there is a need to develop imaging methods that can visualize ROS distribution in wounds and their relationship to the vascular bed and infection.

Luminescence imaging has been used to detect ROS activities in an animal inflammation model.^27^ Several biomaterials and imaging probes have been developed to detect ROS activities *in vivo*.^28^^,^^29^ However, the sensitivity and specificity of these materials/probes for ROS detection are limited. Our and other groups have shown that Lucigenin, luminol, and L-012 are prominent examples of luminescent ROS probes that have been used *in vivo*.^30^ L-012 in particular is well-regarded for its high sensitivity and luminescent output.^31^ Our group has previously used L-012 to detect inflammatory-response-associated ROS activity in acute mouse wounds over time.^32^ In addition, luminescence imaging has been used for noninvasive detection of a wide variety of biomolecular parameters, including peroxynitrite, pH, and reactive nitrogen species.^27^^,^^33^^,^^34^ Luminescence imaging has also been recognized for its potential in medical imaging due to its capacity for noninvasive, time-resolved, detailed molecular imaging.^35^ Despite luminescence imaging’s strong precedence in wound healing and inflammation research, there are no portable imagers capable of performing luminescence imaging on large animals and human subjects.

Currently, *in vivo* luminescence imaging is typically carried out with a large, completely enclosed “black box” imager.^36^^–^^38^ Although these devices provide low noise and high resolution, they are expensive, immovable, and difficult to translate to human or large animal models due to their enclosed design. Several compact luminescence imaging devices have been recently developed to characterize wounds. In 2013, a simple smartphone-based system was developed for burn wound analysis.^39^ This system was easy to use and widely available but was unable to detect luminescence *in vivo* due to the camera’s low sensitivity and lack of a light shield. Near the same time, a ratiometric luminescent lifetime imaging device was fabricated to measure tissue hypoxia consisting of a simple RGB sensor.^40^ This system was inexpensive and able to detect physiological hypoxia in a noninvasive manner but required supplementary fluorescent data and a darkened room to function due to the lack of a light shield. In 2015, a consumer-grade camera was utilized to visualize human chronic wound bioburden using autofluorescent bacteria.^41^ This system was portable and noninvasive but had several downfalls, namely requirement of a light shield, inability to detect luminescence signal due to low camera sensitivity, and reliance on biofluorescent strains of bacteria. There is a need for portable, light-isolated devices capable of imaging luminescence in a wide range of situations. Since luminescent imaging relies on light emitted directly from a probe or bioluminescent tissue, it does not require an excitation light source such as that required for fluorescence imaging. This allows the size and complexity of luminescence imagers to be reduced, which was a general goal of our imager design.

Portable imaging devices are a particularly good fit for several niche medical imaging applications. One notable application is battlefield imaging. Many large imaging devices, such as MRI and CT devices, are impractical to maintain in field hospitals.^42^ Due to this, any medical condition that requires imaging to diagnose must wait to be evaluated until the patient reaches a fully equipped medical facility, preventing the patient from receiving the best standard of care as early as possible. Several portable devices have been developed for different imaging modalities for this purpose. A handheld device has been developed for battlefield applications to diagnose and differentiate between hemorrhagic and ischemic stroke.^43^ Medical imaging in rural areas is another area with the potential to benefit greatly from inexpensive, easy to use, portable imaging devices. Rural hospitals, especially those in third world countries, have limited access to the large and expensive imaging facilities common to a fully outfitted hospital. Unique solutions are in development to circumvent this and provide lifesaving diagnostic services. In rural India and China, retinopathy of prematurity (ROP) is a common cause of infant blindness. When diagnosed promptly, ROP blindness is almost always preventable, but the lack of accessible ocular imaging screening services prevents diagnosis for many individuals. Telemedicine has been investigated as a way to combat this phenomenon, with teams of operators dispatched to at-risk communities in mobile treatment facilities.^44^ Images are acquired and uploaded to a server for remote analysis by a physician.

This report summarizes our development of a portable imager to significantly modify our previous imaging device (generation I), which is only capable of imaging fluorescent and luminescent signals on small animals, but not on humans or large animals.^32^ To overcome these drawbacks, a portable imaging device was fabricated specifically for infection/bioburden detection on large animals via luminescence imaging. We accomplished this by significantly changing the design in several ways, notably substituting the simple laser-cut acrylic frame for a complex computer-aided design (CAD)-designed, 3D-printed imaging chamber. This casing was customized to provide switchable white light/luminescence imaging capabilities for *in vitro* studies as well as large animal wound models. We demonstrate the utility of the device in a porcine model with full-thickness excision wounds incubated with *Psedomonas aeruginosa*. We display the ability of the device to correlate visual discernment of vascularity and ROS activities. Most importantly, our results reveal that wound bioburden can be diagnosed by observing the unique distribution pattern of ROS activity in wounds.

## 2 Materials and Methods

### 2.1 Materials

A CCD camera was used for image acquisition (C10600, Hamamatsu, Bridgewater, New Jersey) with a F1.4/12 mm lens (HR961NCN, Navitar, Rochester, New York). These components were integrated into a light-insulated luminescence imager that was designed using Solidworks CAD software and 3D printed on a commercially available large-format 3D printer (gMax 1.5+, gCreate, Brooklyn, New York) using black polylactic acid (PLA, Hatchbox, Los Angeles, California). Several imager designs were produced for applications ranging from *in vitro* to large animal studies. Light isolation was accomplished using shaped compressible polyurethane (PU) and polyethylene (PE) foam (McMaster-Carr, Douglasville, Georgia).

### 2.2 Device Design and Development

**Fig. 1:**
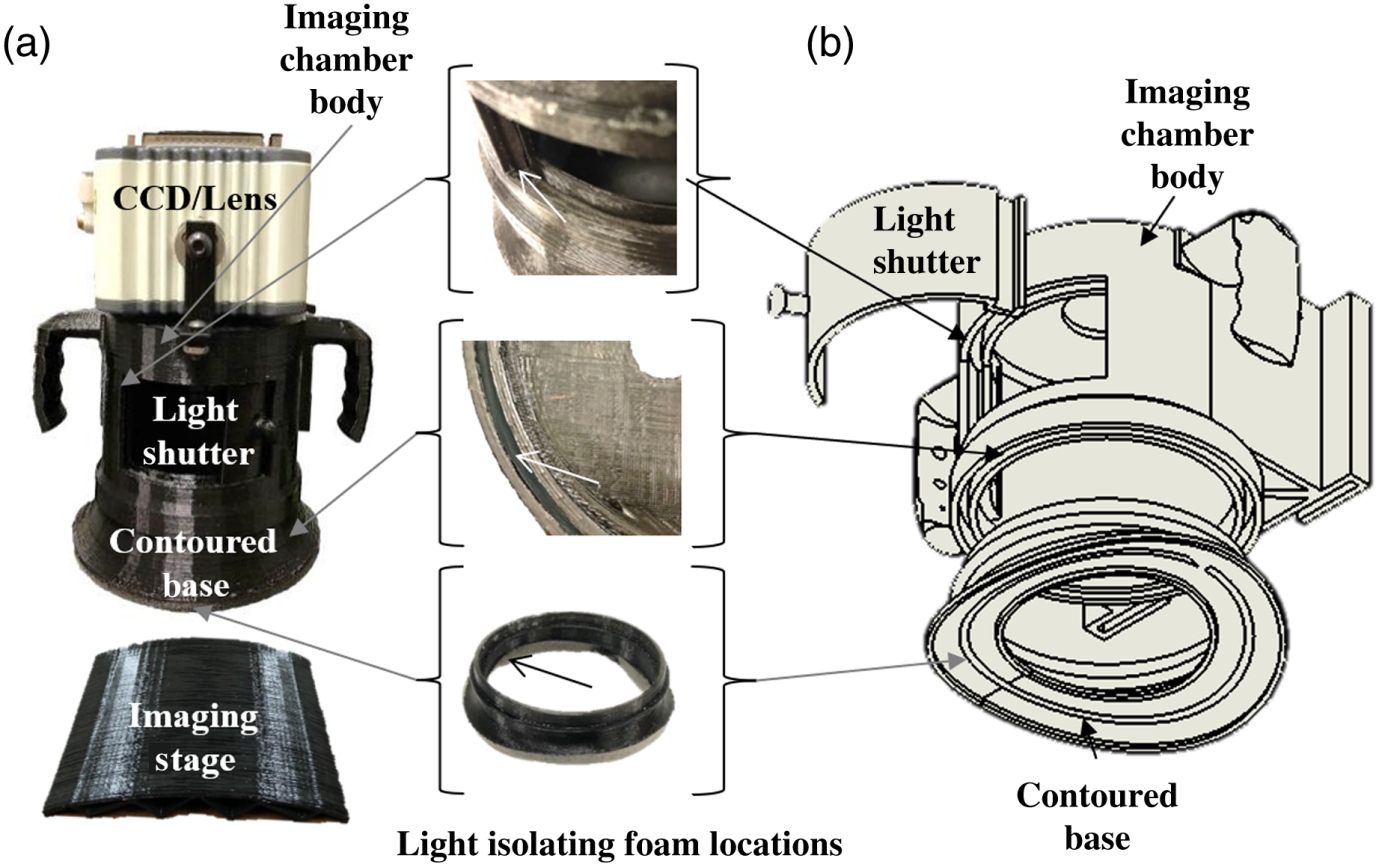
A breakdown of all optical components of the new imaging device is shown here. (a) The device utilizes a multipart design consisting of a CCD, the body of the imaging chamber, an insertable sliding white light portal, and interchangeable contoured bases. Luminescence and white light imaging can be switched between with the adjustable light portal. (b) A CAD drawing depicts the setup of various components associated with the imaging chamber.

The new portable luminescence imager was designed to image wounds on large animal models in a room with low ambient light. Key design parameters for the portable luminescence imager included minimal size, high-luminescence sensitivity, versatility between different imaging scenarios, and complete isolation from ambient light. The device has three major components: a CCD camera, an optical lens, and a custom-designed imaging chamber. A diagram of the imager and its components is shown in Fig. 1(a).

**Fig. 2:**
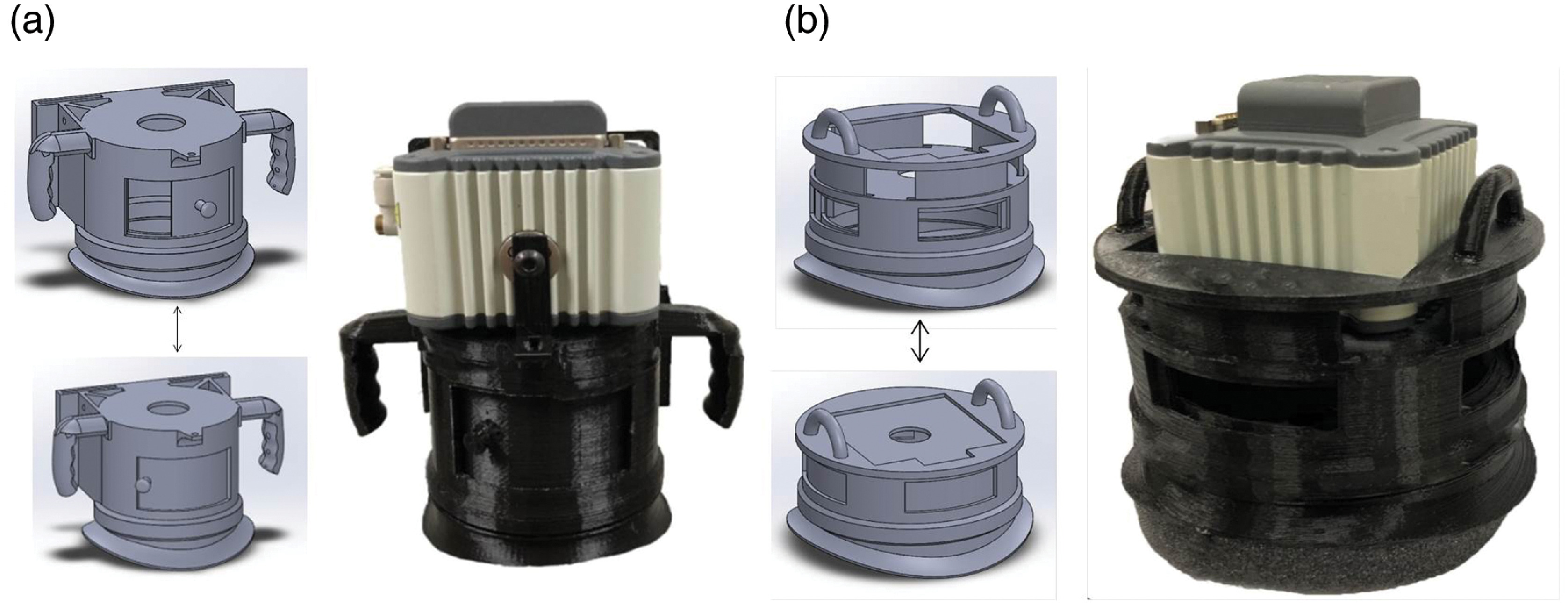
Two designs of a luminescent imager with different working distances. In both designs, white light illumination is provided by a mechanical light shutter. The device can be positioned on the region of interest using a pair of handles or mounted to an articulating arm. (a) An imager for imaging larger wounds (>3  cm diameter) has a 12-cm working distance and (b) an imager for imaging smaller wounds (<3  cm diameter) has a 3-cm working distance.

The imaging chamber was designed and 3D printed for ease-of-use in large animal imaging scenarios. The chamber consists of four main parts, including the main body of the device, a sliding white light shutter, a detachable base designed to contour to the surface of the animal being imaged, and compressible light-isolating foam gaskets. A breakdown of these subparts is shown in Fig. 1(b). The main body of the imaging device mounts to the camera and detachable base. It also contains integrated handles and an on-board standard Video Electronics Standards Association mount to allow for hand positioning or attachment to an articulating arm for hands-free use. The white light shutter allows for rapid exchange between white light and luminescence imaging scenarios with minimal user interaction. The detachable base of the design is contoured to interface directly with the surface of the animal being imaged (Fig. 1). Two revisions of the device were designed with lenses of different working distances to showcase the device’s utility in different large animal imaging scenarios (Fig. 2). The devices allowed different view areas to be captured: 5.14×4  cm [Fig. 2(a)] and 4.75×3.5  cm [Fig. 2(b)] for a zoomed-in or zoomed-out view as desired. In both designs, a base matching the contour of a pig’s back was created. For *in vitro* studies of the effect of animal curvature on imaging, a curved imaging stage with the same contour was also designed and 3D printed. Finally, compressible PU or PE foam gaskets are placed at interfaces to block ambient light. The foam gaskets form the base contours to shield the camera from ambient light for the porcine wound study (Fig. 2).

By comparing physical parameters with a representative commercial imager—the Kodak In Vivo FX Pro—we have determined that the new imager is significantly more compact (new imager: 22×22×25  cm, commercial imager: 104×61×97  cm) and lightweight (new imager: 5 kg, commercial imager: 142 kg) than a standard black-box commercial imaging device.

### 2.3 Image Acquisition and Processing

Image acquisition was handled using software provided by the camera manufacturer (HCImageLive). Images were processed using PortableView, an in-house developed software described previously.^32^ Briefly, this software allows the user to generate manual, semiautomatic, and fully automatic regions of interest (ROIs). It then generates absolute and statistical information from the ROIs and allows the user to superimpose up to three images at a time. For studies involving multiple time points, this allows images to be overlaid and ROIs to be automatically extrapolated to generate ROIs over the same area measured in the initial image. The manual ROI generation tool allows the user to draw freehand or use an adjustable ellipse. The semiautomatic tool allows the user to adjust an intensity threshold within the ROI. The fully automatic tool allows the user to select a contiguous area of interest from which the algorithm will generate an ROI from a user specified iso-intensity level. PortableView utilizes a MATLAB^®^—(Mathworks, Inc., Natick, Massachusetts) based interface with object-oriented C++ wrapped functions (Microsoft Visual Studios) for computational efficiency.

### 2.4 Imager Characterization

The effectiveness of the light isolation strategy was evaluated by measuring background intensity while imaging in room lit with standard incandescent lighting measuring ∼300  lux (20 min, 8×8 binning). Images taken using the same exposure parameters in total darkness were used as a control. Light background was compared with the generation 1 imager developed in our previous work and with the industry-standard Kodak In Vivo FX Pro.

The sensitivity limit of detection (LOD) of the portable imager’s ability to detect ROS-associated luminescence *in vitro* was compared with the Kodak using a calibration curve.^45^ Two hundred μL of five concentrations of $H_{2}O_{2}$ (2500, 625, 156.25, 39.1, and 0  μM) was added in triplicate for each concentration to wells of a 96-well plate. Twenty μL L-012 (15  mg/mL) were added to each well. The LOD and sensitivity of the imager’s luminescence detection were analyzed. LOD was defined as the lowest concentration each machine is capable of detecting and was calculated using the formula:^46^

$$\mathrm{LOD}=\frac{3\sigma }{k},$$

where σ is the standard deviation of the control and κ is the slope of the linear curve.

Sensitivity was determined by the slope of the linear line of best fit of the standard curve, as previously defined.^47^^,^^48^

The ability of the imager to detect different concentrations of ROS using a new ROS film was characterized by applying six different concentrations of $H_{2}O_{2}$ to L-012-based ROS sensing films (provided by Progenitec Inc., Arlington, Texas; 50  μL; 0, 1, 5, 10, 25, 50, and 100  μM). The established ROS-sensing probe L-012 was used as a control, with 20  μL of L-012 (15  mg/mL) mixed with 200  μL of $H_{2}O_{2}$ in the wells of a 96-well plate. Sensitivity and LOD were calculated as described above.

Homogeneity was determined on surfaces of different curvatures by arranging consistent droplets of $H_{2}O_{2}$ (100 mM, 20  μL) in a matrix pattern on the imaging stage and injecting L-012 in each droplet directly prior to imaging (5  μL, 48.3 mM). Luminescent signals were acquired for 12 min at 8×8 binning, and consistency at the center and edges of the stage was quantified.

### 2.5 Animal Study

The ability of the imaging modality to detect ROS in wounds *in vivo* was examined a porcine wound model.^9^^,^^49^ This animal protocol was approved and all animals were cared for according to the standard guidelines approved by the Animal Care and Use Committee at the University of Texas Southwestern Medical Center at Dallas in accordance with the Animal Welfare Act and Guide for the Care and Use of Laboratory Animals.

The imager’s ability to visualize both vascularization and ROS distribution in healing wounds was proven using a porcine wound healing model. Vascularization was observed under white light illumination and ROS observed using luminescence. An established excisional wound model was used.^49^ Briefly, female pigs with a weight between 90 and 120 lbs were used in this investigation. Aseptic technique was used to place six full-thickness wounds (3-cm diameter, 2 wounds per treatment group per animal) on the dorso-lateral surfaces of the animal. After wounding, two wounds per animal were inoculated with ∼2000 colony-forming units of *Pseudomonas aeruginosa* (American Type Culture Collection 27853) per wound. All wounds were packed with gauze and covered with Tegaderm (3M, St. Paul, Minnesota). Dressings were changed at days 3, 7, 10, 14, 17, and 21. Before dressing changes, a circular section of ROS-sensing film was applied to the wound prior to wound imaging. The imager was then positioned over the wound and the light portal was moved to the open position. A white light image was then acquired for blood vessel visualization. Finally, the light portal was moved to the closed position and a luminescence image was acquired. Wounds images were analyzed using PortableView software as described above. The relationship between blood vessel location and ROS distribution was investigated. Additionally, the change in average intensity over time was compared between infected and uninfected wounds. Finally, the difference in infected versus uninfected wound ROS cluster number and average integrated density (defined as the sum of cluster intensity values divided by cluster area) was analyzed.

### 2.6 Statistical Analysis

All values are reported as average±standard deviation. ROS levels and luminescent intensities were correlated using linear regression, with fit measured by the coefficient of determination ($R^{2}$). Significant difference in ROS-associated intensity between vascular- and capillary-associated areas was determined with a z-transformation test as described previously.^50^ Briefly, a test statistic ($Z_{s}$) was calculated from transformed p-values ($Z_{i}$) from k paired, one-tailed t-tests computed for each wound according to the equation:

$$Z_{s}=\frac{\sum_{i}^{k}Z_{i}}{\sqrt{k}},\;Z_{i}=\tanh^{-1}\;P_{i}.$$

This value was then compared to $Z_{\mathrm{crit}}$ values for a 95% confidence interval (±1.96). The significance in the difference between average ROS levels over time between infected and uninfected wounds was determined with a paired Student’s t-test with an α of 0.05. Significant difference between treatment groups in all other studies was determined with an unpaired Student’s t-test with an α of 0.05.

## 3 Results and Discussion

### 3.1 Imager Characterization

**Fig. 3:**
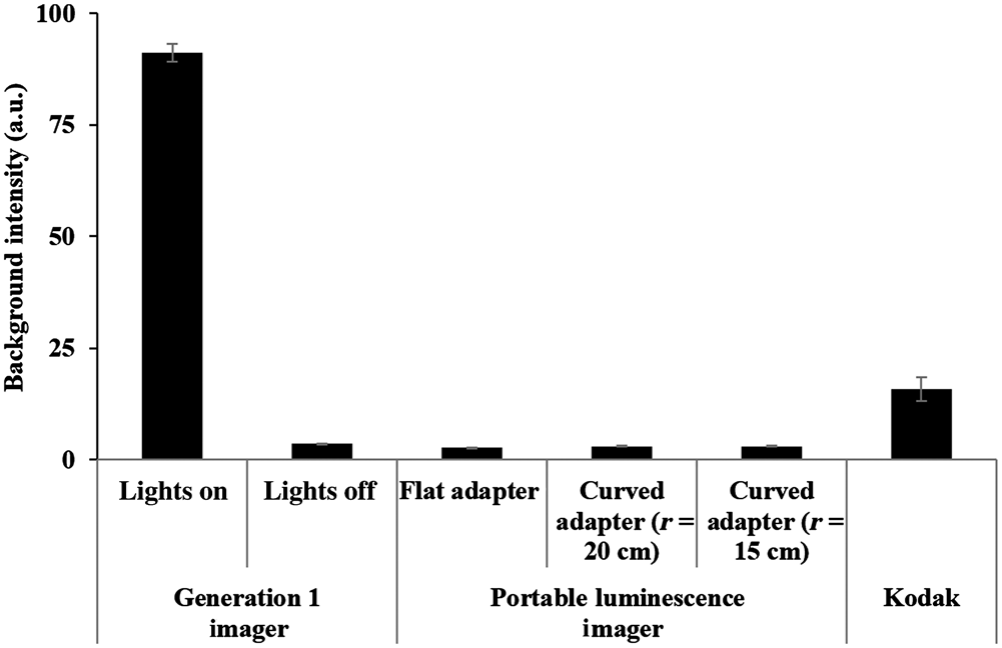
Comparison of light isolation efficiency between the generation 1 imager, the new portable luminescence imager, the Kodak In-Vivo FX Pro, and contoured imager-base designs. Images were taken under ambient light with the following imaging conditions: 8×8 binning, 4-min exposure. All bases were found to have acceptable background levels (below 10 a.u.) and represent a significant improvement over the previous imager’s background levels. The new luminescence imager was also found to have significantly lower background than the state-of-the-art Kodak imager.

The ability of the novel imager to isolate the imaging environment from ambient light in a lit room was compared to the light isolation solution from the previous fluorescent/luminescent imaging device (blackout fabric). Several adapters were used with varying curvatures (flat versus curved with radius of 25, 20, or 15 cm) to assess the effect of a curved stage interface on light isolation. Images were taken at exposure times of 4 min and 8×8 binning in a lit room and background signal was quantified (Fig. 3).

The new luminescence imager was shown to be light-tight in a procedure room lit with typical standard incandescent overhead lighting (∼300  lux) for both flat and contoured surfaces, with background values equivalent to total darkness in the imaging room. This was a background intensity reduction of ∼30 times from the generation I imager designed for both luminescence and fluorescence, which enabled the detection of even weak luminescence signals without interference in the new device. The new imager was also found to produce ∼5 times lower background than the Kodak imager, establishing its excellent performance in background reduction even compared to commercially available devices.

**Fig. 4:**
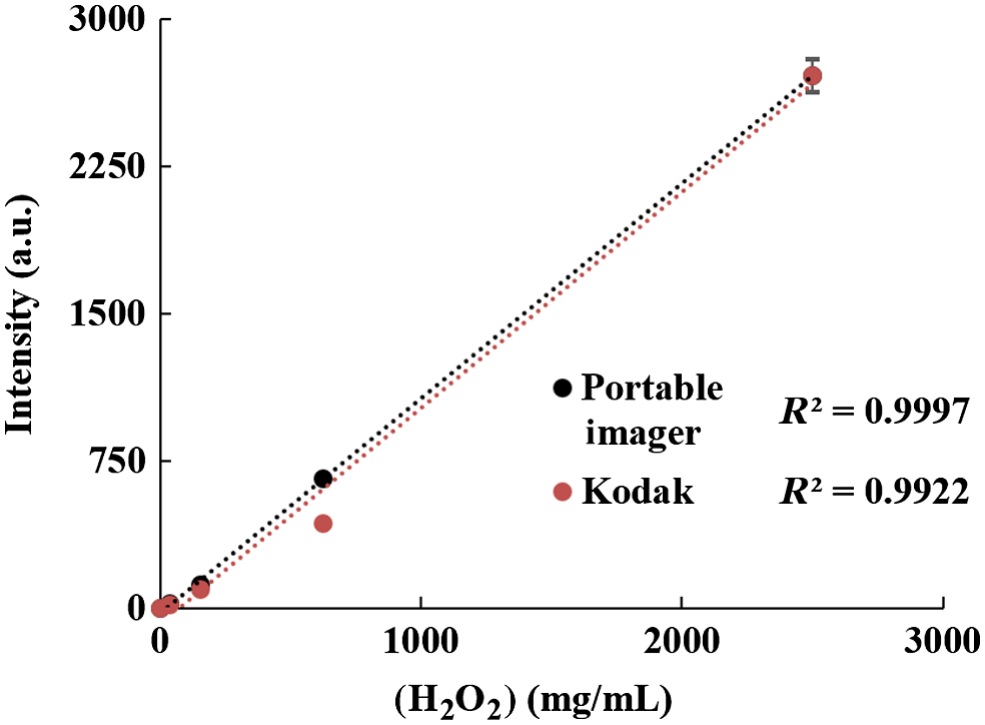
The sensitivity and LOD of the portable imager were compared with the Kodak imager using the luminescent ROS probe L-012. The new imaging device was found to produce luminescence sensitivity and LOD comparable to the Kodak imager.

The LOD and sensitivity of the portable imager’s response to luminescent signals were compared with the Kodak imager using a calibration curve *in vitro* (Fig. 4). Fourfold serial dilutions of $H_{2}O_{2}$ were added to wells of a 96-well plate in triplicate. L-012 was added to each well immediately prior to imaging (20  μL, 15  mg/mL).

The sensitivities of the two imaging devices were found to be 1.0941±0.140  a.u./μM for the portable imager and 1.0996±0.145  a.u./μM for the Kodak. The LOD for the portable imager was calculated to be 0.9  μM compared to the Kodak’s 18  μM, suggesting that the portable imager can detect slightly lower concentrations of ROS in this scenario than the Kodak imager. In addition, both devices were found to produce an excellent linear relationship between ROS concentration change and luminescence. These results support that both imagers are capable of quantifying low concentrations of luminescent signal with excellent sensitivity.

**Fig. 5:**
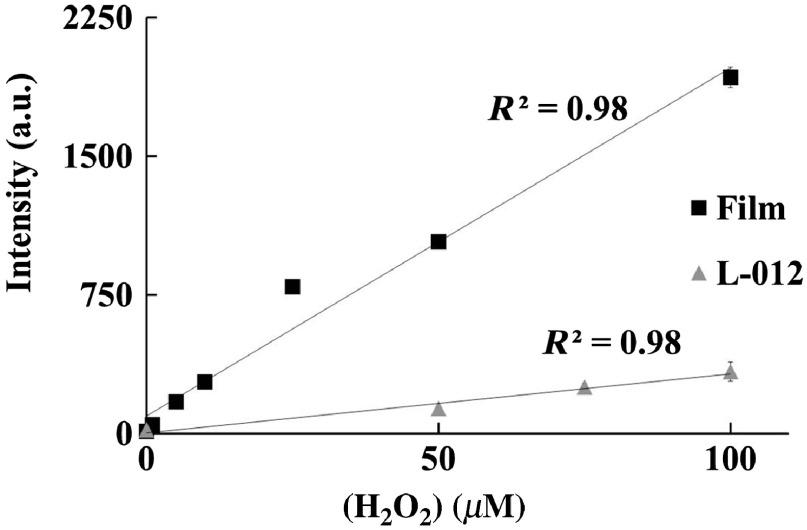
The ability of the luminescence imager to quantify ROS was tested *in vitro* using ROS-sensing film or the probe L-012. The imager was found to be capable of detecting physiologically relevant concentrations of ROS (<5  μM). ROS-sensing film was found to have significantly lower LOD and higher sensitivity than L-012.

The sensitivity and LOD of the relationship between ROS concentration and ROS-sensing film luminescence were characterized *in vitro* (Fig. 5). Several concentrations of $H_{2}O_{2}$ within the physiologically relevant range (5  μL; 0, 1, 5, 10, 25, 50, and 100  μM) were applied to luminescing ROS-sensing film or treated with the luminescent probe L-012. Exposures were taken immediately after treatment.

The imager was found to correlate luminescent signal and ROS concentration with a robust linear relationship for both the ROS sensing film and L-012 ($R^{2}=0.98$). The mechanism governing the higher sensitivity and lower LOD of the ROS sensing film over those of L-012 solution has yet to be determined. It is possible that by adding L-012 into the wound dressing, the ROS-sensing film was packed with a large number of L-012 probes adjacent to each other. Such dense arrangement of L-012 probes may contribute to signal amplification. The imaging system also showed high sensitivity ($k_{\text{film}}=18.9\pm 0.4\;\mathrm{a.u.}/\mu M$ and $k_{L\text{-}012}=3.2\pm 0.2\;\mathrm{a.u.}/\mu M$) and was able to detect low concentrations (${\mathrm{LOD}}_{\text{film}}=0.11\;\mu M$, ${\mathrm{LOD}}_{L\text{-}012}=1.2\;\mu M$). The results support that the imager has excellent luminescence sensing properties sufficient for *in vivo* imaging.

**Fig. 6:**
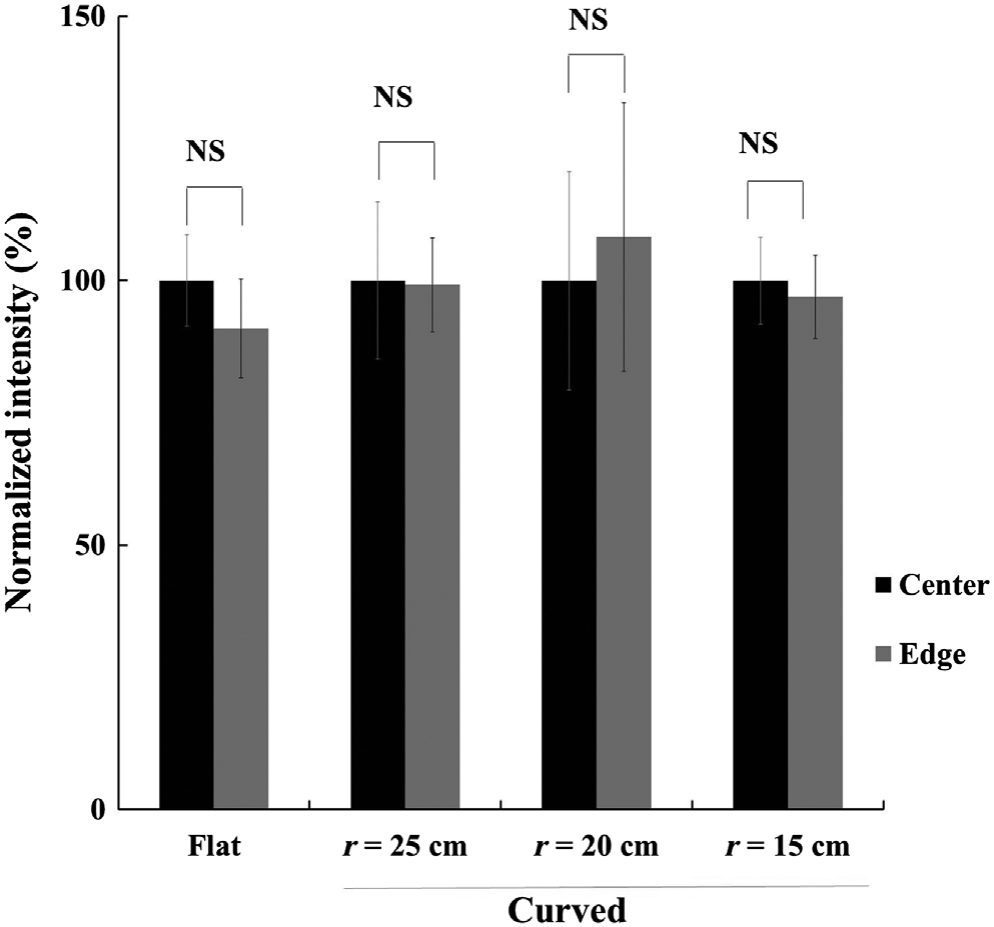
The effect of base contours on luminescence homogeneity was assessed by quantifying a matrix of $H_{2}O_{2}$ mixed with the luminescent ROS probe L-012 arrayed over the view area of each stage with various curvatures. We statistically analyzed the luminescent intensities between edge and center (calibrated as 100%) on different platforms. We found that curvature has no statistically significant influence on the luminescence readings by comparing the readings at the center and edge of the base plate.

The homogeneity of luminescent signals on surfaces with varying degrees of curvature was investigated to identify any significant decrease or variation in intensity with a consistent ROS signal across the field of view. Curvature was defined as the inscribed circular radius of the base. Droplets of $H_{2}O_{2}$ (100 mM, 20  μL) were arranged in a matrix pattern on the imaging surface. Before imaging, L-012 (5  μL, 48.3 mM) was added to each droplet. We found that there is no significant difference in luminescent intensities detected by imagers with flat and curved bases. In addition, the extent of curvature has no significant influence on luminescent intensity at different regions from the center of the imaging area to the edge (Fig. 6).

These results support that the imager with curved imaging bases can be used for imaging curved objectives (such as porcine back or human arms and legs). Although a curved imaging stage is not exactly equivalent to the natural curvature found in living subjects, the lack of significant intensity variation between high and low points of a curved stage simulating biological curvature in this study provides strong evidence that this imaging device can be used *in vivo* without the necessity to perform additional intensity calibration.

### 3.2 Porcine Wound Imaging

**Fig. 7:**
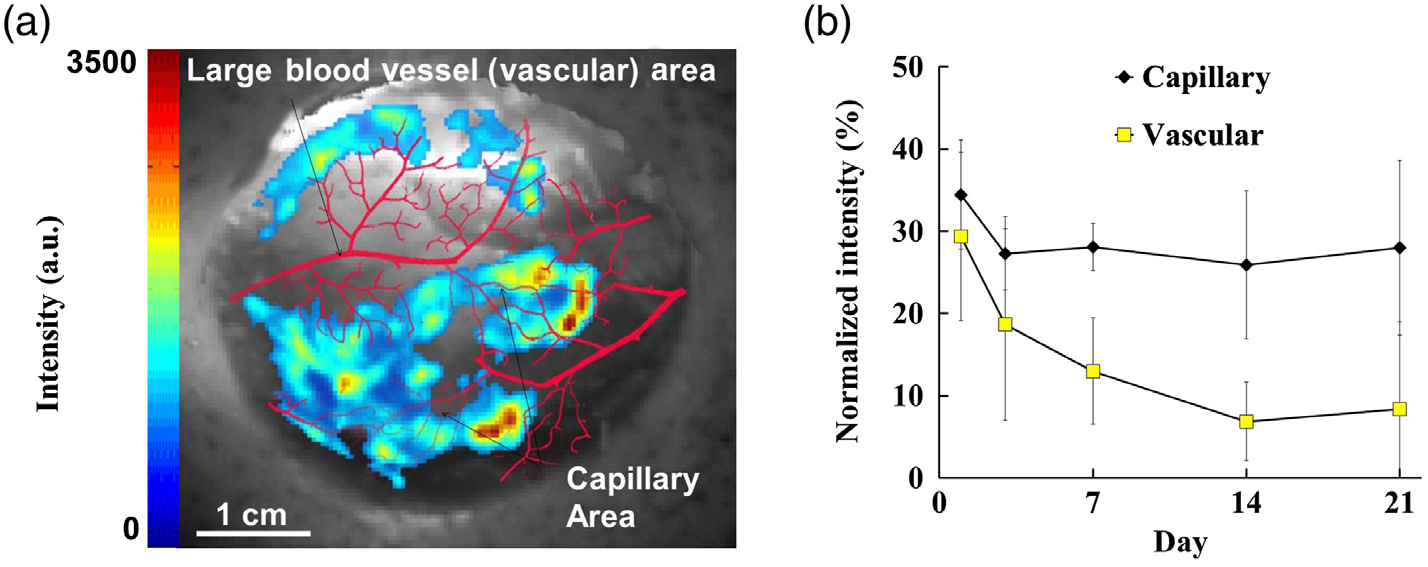
Luminescence (ROS) and white light (blood vessels) images were captured for each wound. The spatial relations between ROS activity and vasculature are shown in a representative wound. (a) Luminescent ROS images were overlaid on white light images of the wound bed to reveal the relationship between ROS activity and vasculature (shown enhanced in red). (b) Higher intensity was found to inversely correlate with large blood vessel location. This trend was found to be stable over time and statistically significant ($Z_{s}=0.44$ and $Z_{\text{crit}}=\pm 1.96$), with ROS intensity in capillary associated areas of the wound roughly double than areas associated with large vessels on average.

The ability of the portable imaging device to correlate ROS distribution via luminescence and blood vessel location via white-light imaging was investigated. Luminescent and white light imaging pairs were used to create overlaid images of ROS levels and blood vessel distribution. Wounds with clearly visible blood vessels at day 1 were analyzed over the 21-day study (n=7). As shown in the representative image [Fig. 7(a)] and graphical comparison [Fig. 6(b)] of the change in intensity in wound areas, we find the majority of ROS activities are found in areas near capillaries and far away from large visible vessels (0.5-mm diameter). Large vessels were enhanced in red for increased visibility.

By analyzing the luminescent intensity and vasculature distribution [Fig. 7(b)], we find that ROS intensity is significantly higher in areas of the wound ≥2.5  mm away from large visible blood vessels ($Z_{s}=0.44$ and $Z_{\text{crit}}=\pm 1.96$). The results support that ROS activity is highest in capillary-dense areas of the wound bed, rather than in the vicinity of larger, visible vessels. This is consistent with expectations and current understanding of ROS production in healing wounds.

**Fig. 8:**
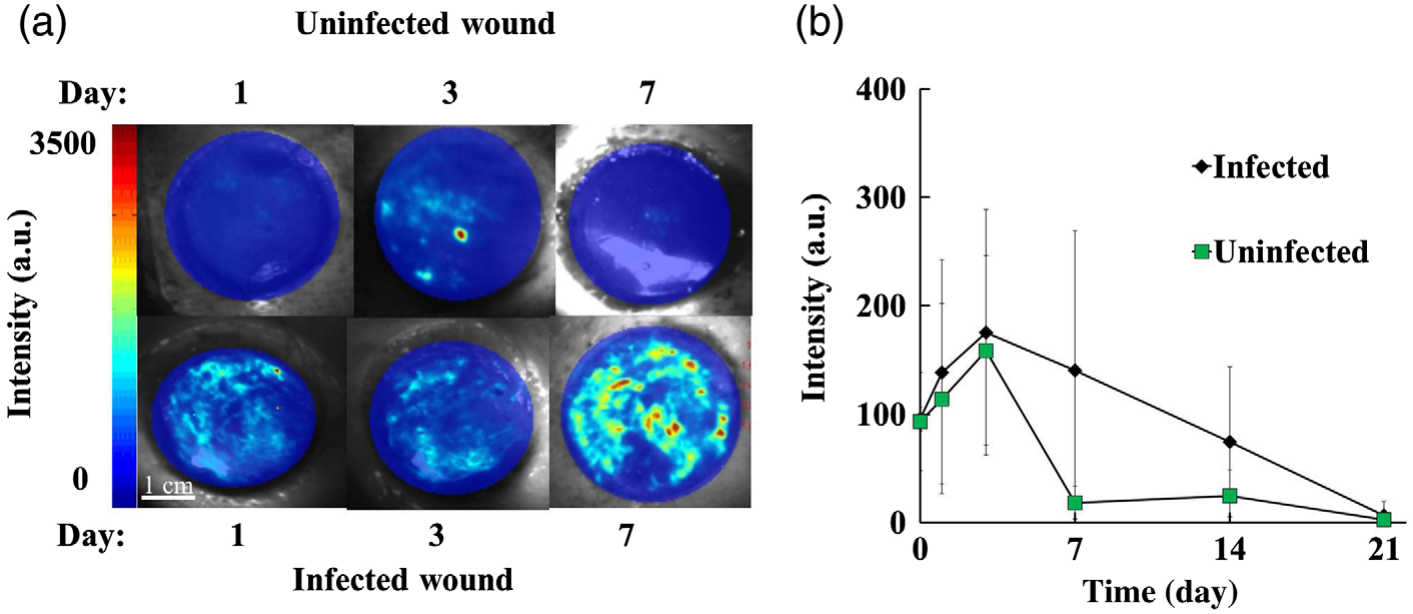
The change in luminescent intensity of infected and uninfected wounds was documented for 21 days with the luminescence imaging system. (a) A representative panel of overlaid luminescence/white light images of infected and uninfected wounds over the first seven days is shown. (b) The line graph of average wound ROS-associated intensity changes over time shows that there are significantly higher ROS levels in infected wounds over the duration of the study, especially on days 7 and 14, than in control wounds (p=0.019).

We also compared the luminescent signals between infected and uninfected wounds over 21 days. A representative image panel showing uninfected and infected wound ROS levels at 1, 3, and 7 days is shown in Fig. 8(a).

Wounds with high bioburden were found to maintain high ROS levels longer than uninfected wounds (p=0.019). This effect was most prominent from day 3 to day 21, with much of the ROS intensity for uninfected wounds remaining very low after 1 week [Fig. 8(b)]. ROS levels in infected wounds were found to peak between day 3 and day 10, whereas uninfected wounds declined quickly after day 3. This finding supports established knowledge of ROS responses in normal and infected wound healing in a new two-dimensional (2-D) model.

**Fig. 9:**
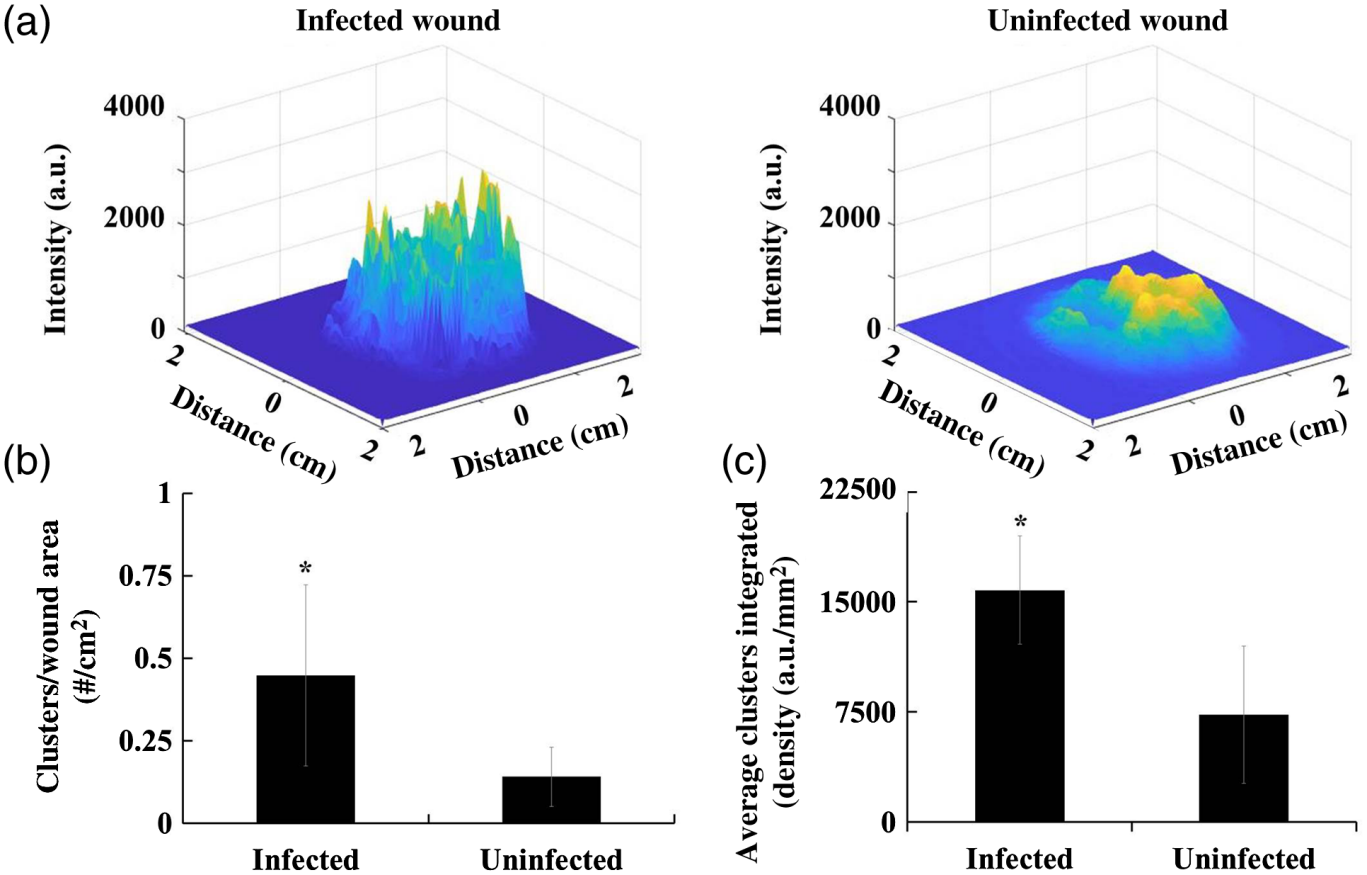
ROS distributions in infected and control wounds were compared. (a) Wound images for infected and noninfected wounds were analyzed in MATLAB and surface plots were generated. Representative surface plots for both wound categories are shown here, displaying more numerous and sharper peaks in infected versus uninfected wounds. (b) By comparing both groups of wounds, we find that infected wounds have larger numbers of high-intensity ROS activity clusters than control wounds (p=0.04). (c) ROS cluster areas were observed to have higher integrated density per square millimeter in infected wounds than in uninfected wounds, supporting the observed sharper ROS intensity peaks in infected wounds (p=0.014).

In addition to increased ROS activity, we have also observed that infected wounds have more clustered high-intensity ROS areas (Fig. 9). We compared the ROS distribution patterns between infected and uninfected wounds, selecting one time point with the maximum ROS signal for each wound for consistency. To visualize this, surface plots were generated to compare peak height and size between infected and uninfected wounds [Fig. 9(a)]. In MATLAB, intensity clusters above a 70% maximum intensity threshold were detected and quantified for infected and uninfected wounds. The number of clusters in the wound area was then defined and compared between the two treatment groups [Fig. 9(b)]. In addition, average integrated density was compared between the two treatment groups [Fig. 9(c)].

The imaging system discovered a more clustered distribution of ROS over the wound bed in infected than in uninfected wounds, with ∼3 times the number of peaks occurring per ${\mathrm{cm}}^{2}$ in infected wounds [Fig. 8(b), p=0.04]. It was also observed that cluster peaks of infected wounds were significantly higher and sharper compared to the lower, broader peaks of uninfected wounds. Finally, by comparing the average integrated densities of cluster areas, we find that infected wounds have significantly higher cumulative intensity signals in ROS cluster areas than noninfected wounds (p=0.014). These results suggest a good relationship between ROS activity distribution, specifically cluster number and area-averaged integrated density, and bacterial colonization in wounds. These findings point to a less homogeneous and “patchier” ROS distribution in wounds with a high bacterial bioburden. Overall results suggest that the “patchier” ROS distribution can be used as an “imaging signature” for wound bioburden diagnosis.

## 4 Discussion

There is a robust demand for imaging modalities that can visualize molecular parameters of wound healing in a real-time, noninvasive manner. Although many imaging methods have been developed with the capability to image wounds, many of them are too large to transport easily and require expensive instrumentation. In addition, imaging devices designed for *in vivo* measurement of luminescent signals are completely enclosed to prevent light contamination, which prevents the imaging of large animals and precludes interacting with the subject over the course of imaging. This report describes the refinement of our generation 1 imager design into a portable imager optimized for detecting luminescence, particularly associated with ROS in skin wounds. Similar to the previously described imager design, this luminescence imager has many advantages over large commercial imagers, including increased portability, decreased size, and ability to image in a much wider range of scenarios due to its open design. The device is extremely lightweight and portable, allowing it to be transported between imaging locations and positioned by one of several mounting options or by hand with ease. This presents significant advantages over commercially available black-box imagers, especially in niche applications such as rural clinic or battlefield medical imaging. Additionally, this device was found to have almost identical luminescence imaging performance to these commercial devices *in vitro*. A two-part design allows the sample-interfacing lower portion of the imaging device to be swapped with differently contoured parts for imaging in a wide range of scenarios, ranging from *in vitro* imaging on a tabletop to imaging the uneven surface of a large animal in a lit procedure room. The device has drastically reduced many issues associated with the early prototype, such as limited mechanical strength, a lack of adjustability, and inconsistencies in white light illumination.

Our results support that the new imager is able to detect ROS activity and bacterial bioburden in large animal wounds. Neutrophils have been found to be the major cell type responsible for ROS production.^31^ Furthermore, ROS distribution and perhaps activated neutrophils were found to accumulate more prominently at capillary-infused areas of wounds rather than areas adjacent to large vessels. This phenomenon is supported by the fact that the primary cellular producers of ROS in wounds, neutrophils and other inflammatory cells, migrate into the wound through postcapillary venules.^16^ This trend was also consistent over the course of the study, with areas of the wound not populated with large visible vessels consistently expressing almost double the intensity of areas near these vessels. This represents the first visual observation of the relationship between ROS and blood vessel distribution in wound healing with the support of many previous biological findings in inflammatory cell recruitment and associated redox responses.^15^^,^^20^^,^^51^

Wound bioburden, the result of bacterial colonization in wounds, has significant impact on wound healing and care. Wound bioburden/infection is typically diagnosed with visual observation and confirmed by laboratory culture, both methods with many drawbacks.^7^^,^^52^ Briefly, visual observation may not be accurate for detecting bioburden/infection and laboratory culture is time consuming. Our study has shown that the combination of the developed luminescent imager and ROS probes can be used for real-time wound bioburden/infection detection. Using our imaging system, we find that infected wounds have significantly more clustered distributions of ROS than uninfected wounds. This is consistent with previous findings that show that ROS is highly correlated with local presence of bacteria.^16^^,^^17^ The previously described clustering behavior of bacteria likely also contributes to this phenomenon.^53^ This finding represents the first known association of 2-D ROS distribution and wound bioburden/infection status *in vivo* and may pave the way for new methods of diagnosing bioburden/infection in clinical settings.
